# Intraortic balloon pump use in cardiac surgery: analysis of data from the ARIAM Registry of Cardiac Surgery

**DOI:** 10.1186/cc14278

**Published:** 2015-03-16

**Authors:** J Muñoz-Bono, MD Delgado-Amaya, E Curiel-Balsera, C Joya-Montosa, G Quesada-García

**Affiliations:** 1 Hospital Regional de Málaga, Spain

## Introduction

The aim of the study is to analyze IABP use in patients undergoing cardiac surgery included in the ARIAM Registry of Cardiac Surgery.

## Methods

An observational, retrospective, multicenter study of all patients undergoing cardiac surgery included in the ARIAMANDALUCIA database of Cardiac Surgery from March 2008 to July 2012. We used the chi-square test and Student *t *test as needed, establishing the level of statistical significance at 95%.

## Results

Of the 8,026 patients who underwent cardiac surgery during the study period, BCIAO was implemented in 358 (4.5%) of them. In total, 65.4% were male. Surgical times in those patients where IABP was implanted were 146 ± 81 minutes and 90 ± 66 minutes (cardiopulmonary and aortic clamping times, respectively). The in-surgery room mortality was 4.7%, 30-day mortality in these patients was 40.2%. Patients in whom IABP was implanted had a mortality rate eight times higher than those who did not require it during surgery or postoperatively (40.2% vs. 8.4%, *P *= 0.0001. OR 8.1, 95% CI (6.4 to 10.3)). Besides mortality was higher, the later IABP was implanted the higher the mortality rate was (29.6% of the preoperative, 44.2% of surgical and 54.4% of those starting in ICU, *P *= 0.015). The ICU length of stay was 9 ± 22 days while the hospital length of stay was 21 ± 28 days. In patients who needed IABP, the ICU stay was higher than for those who did not need it (9 ± 22 vs. 5 ± 10 days, *P *= 0.002) whereas there was no difference in hospital stay (21 ± 28 vs. 20 ± 24 days, *P *= 0.054).

## Conclusion

The intra-aortic balloon pump was used by 4.5% of surgeries performed during the study period and in patients with an increased risk of perioperative complications, estimated by EuroSCORE. ICU length of stay was higher in patients requiring IABP, with no differences in overall hospital stay. Mortality rate was 40% higher, and increases with the delay in the implantation.

